# Target Therapy in Thyroid Cancer: Current Challenge in Clinical Use of Tyrosine Kinase Inhibitors and Management of Side Effects

**DOI:** 10.3389/fendo.2022.860671

**Published:** 2022-07-08

**Authors:** Ivana Puliafito, Francesca Esposito, Angela Prestifilippo, Stefania Marchisotta, Dorotea Sciacca, Maria Paola Vitale, Dario Giuffrida

**Affiliations:** ^1^ Medical Oncology Unit, Istituto Oncologico del Mediterraneo SpA, Viagrande, Italy; ^2^ IOM Ricerca Srl, Viagrande, Italy; ^3^ Internal Medicine Unit, Giuseppe Giglio Foundation, Cefalù, Italy; ^4^ Hospital Pharmacy Unit, Istituto Oncologico del Mediterraneo SpA, Viagrande, Italy

**Keywords:** thyroid cancer, target therapy, adverse events, tyrosine kinase inhibitors, multikinase inhibitors

## Abstract

Thyroid cancer (TC) is the most common endocrine malignancy. TC is classified as differentiated TC (DTC), which includes papillary and follicular subtypes and Hürthle cell variants, medullary TC (MTC), anaplastic TC (ATC), and poorly differentiated TC (PDTC). The standard of care in DTC consists of surgery together with radioactive iodine (^131^I) therapy and thyroid hormone, but patients with MTC do not benefit from ^131^I therapy. Patients with advanced TC resistant to ^131^I treatment (RAI-R) have no chance of cure, as well as patients affected by ATC and progressive MTC, in which conventional therapy plays only a palliative role, representing, until a few years ago, an urgent unmet need. In the last decade, a better understanding of molecular pathways involved in the tumorigenesis of specific histopathological subtypes of TC has led to develop tyrosine kinase inhibitors (TKIs). TKIs represent a valid treatment in progressive advanced disease and were tested in all subtypes of TC, highlighting the need to improve progression-free survival. However, treatments using these novel therapeutics are often accompanied by side effects that required optimal management to minimize their toxicities and thereby enable patients who show benefit to continue treatment and obtain maximal clinical efficacy. The goal of this overview is to provide an update on the current use of the main drugs recently studied for advanced TC and the management of the adverse events.

## 1 Introduction

Thyroid cancer (TC) represents the most common endocrine malignancy. According to GLOBOCAN 2020 data, TC results as the eighth most diagnosed malignancy in the world in 2020, with a low death rate compared to other malignancies (43,646 deaths on 586,202 new cases diagnosed in both sexes and all ages) (1). The incidence of TC by sex showed a higher prevalence in women, ranging from 3- to 4-fold higher than in men (1). The incidence of TC increased in the last two decades: starting from 2000, the rate of new cases grew exponentially year by year, reaching a plateau in the last decade (2). This increase is likely due to the use of sensitive neck ultrasound, which allows to detect incidentally small intrathyroidal tumors.

The thyroid gland comprises both follicular cells and medullary parafollicular cells: follicular cells express a sodium–iodide symporter (NIS) for iodine entry, while medullary parafollicular cells are neuroendocrine in origin and are also known as “C” cells.

The current version of TC classification was introduced in 2017 (3). TC classification is based on the histopathological subtype of cancer cells: differentiated TC (DTC) arises from the follicular cells of the thyroid; it is the most common form, representing more than 90% of all diagnosed TC cases. DTC is subclassified as *i*) papillary TC (PTC), which is the prevalent form of DTC, occurring in 80% of cases; *ii*) follicular TC (FTC), occurring in 10%–20% of DTC; *iii*) Hürthle cell variants (2%–8% of diagnosed DTC). Medullary TC (MTC) develops after a malignant transformation of the neuroendocrine “C” cells; it accounts for about 2% of all TC cases. MTC represents a unique TC; it was recognized that the tumor occurred either sporadically or in a hereditary form as a component of type 2 multiple endocrine neoplasia (MEN) syndromes, MEN2A and MEN2B, and the related syndrome, familial MTC (FMTC) (4). Anaplastic TC (ATC) is the undifferentiated form and represents the remaining 2% of all TC cases. In 1983, Sakamoto *et al.* proposed a new entity, poorly differentiated TC (PDTC), and described its clinicopathological features (5). In 2004, the *WHO Classification of Tumours of Endocrine Organs* recognized PDTC as a new subtype of TC (6), representing the bridge between DTC and ATC, with poor differentiation and greater aggressiveness reflecting a poor overall survival (OS). The common clinical features of PDTC are increased local growth and distant metastases (7).

TC in the pediatric population is rare. The most frequent TC type occurring in the pediatric population is PTC (approximately 90% of pediatric TC), followed by FTC (~10%), MTC (3–5%), and rarely ATC and PDTC. PTC is frequently associated with more extensive extrathyroidal disease (8).

The current treatment for TC consists of surgery together with radioactive iodine (^131^I) therapy (in those phenotypes showing avidity for iodine due to turnout of NIS) and thyroid hormone (9).

DTC is usually curable with surgery and ^131^I therapy, with an indolent disease course in both PTC and FTC showing a high 10-year survival in patients with no local progression and absent local/distant metastases (about 85%). Nevertheless, additional treatments are required in patients with local recurrence and/or distant metastases. In some cases, patients with aggressive DTC became refractory to ^131^I treatment; these patients have a poor overall prognosis and an OS rate of less than 15% at 10 years (10, 11).

In contrast to DTC patients, patients with MTC do not benefit from ^131^I therapy, because they lack the NIS necessary to incorporate ^131^I within the cells (4). Therefore, almost always patients with MTC undergo initial surgery, in most cases associated with lymphadenectomy. In case of persistent or residual local disease, external beam radiation to the neck may be used (4).

Actually, ATC is the most aggressive form of TC, associated with high mortality. Since the ATC rapidly degenerates into progressive malignancy, its cure represents an urgent unmet need.

Until a few years ago, patients with advanced TC resistant to ^131^I treatment (RAI-R) had no chance of cure, as well as patients affected by ATC and progressive MTC. Conventional therapy plays only a palliative role in these patients, because of its poor efficacy, resulting in no prolongation of survival in the use of a single therapeutic agent or in combination with other drugs (12).

In the last decade, a better understanding of molecular pathways involved in the tumorigenesis of specific histopathological subtypes of TC has led to develop tyrosine kinase (TK) inhibitors (TKIs), a novel class of compounds targeting these pathways, implicated in the proliferation and neoangiogenesis of TC.

The objective of this overview is to provide an update on TC treatments using TKIs, mentioning clinical trials in which their use resulted in considerable progression-free survival (PFS). A discussion of the most common toxicities reported will be provided, focusing on the management of the side effects of TKIs in TC.

## 2 Molecular Alterations in Thyroid Cancer

**Figure 1:**
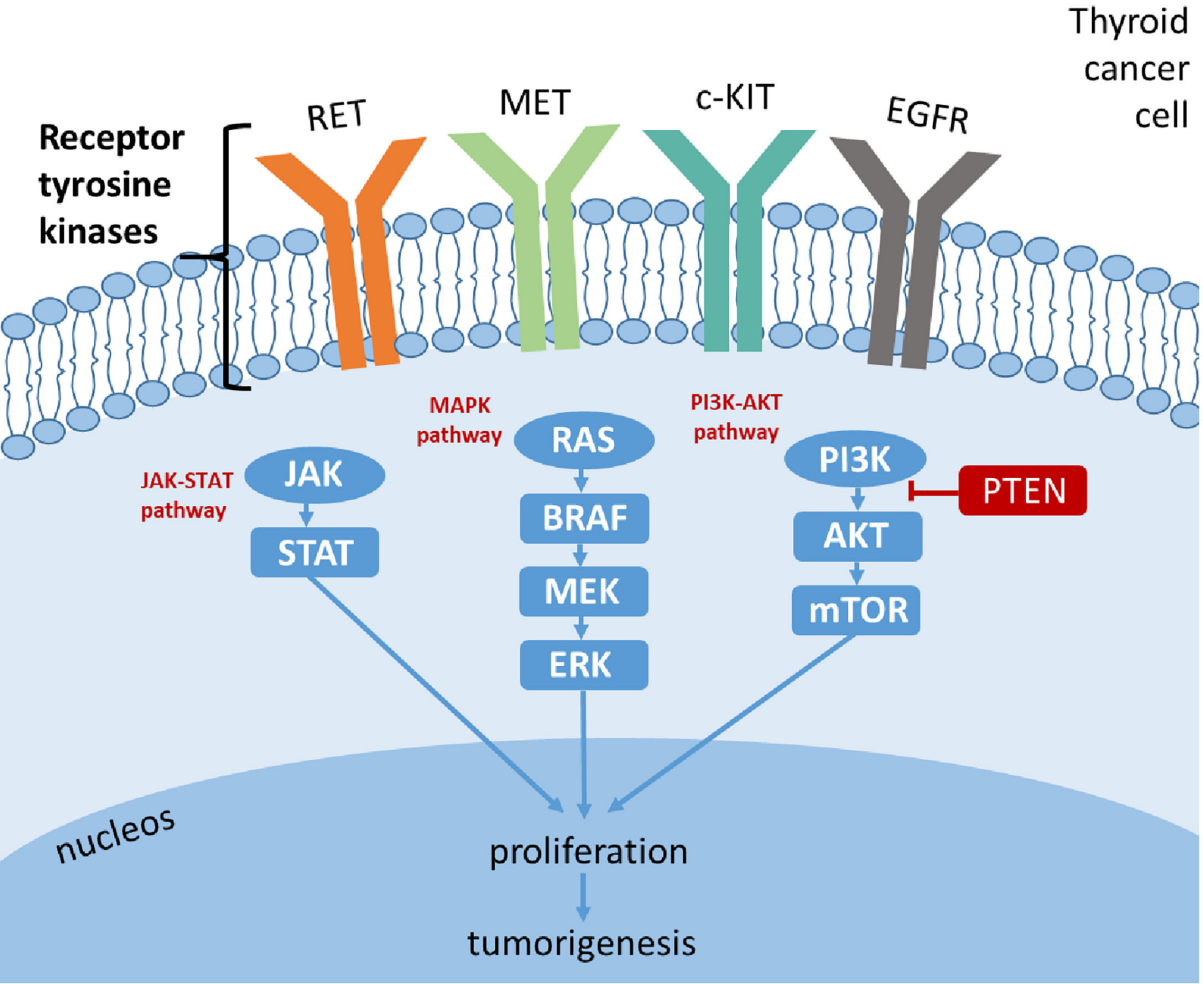
Signaling pathways in thyroid cancer.

TKs have emerged as a key pharmacological target in oncology (13). TKs are key enzymes involved in the control of mitogenic signal transduction through phosphorylation/dephosphorylation of many intracellular proteins: these proteins are responsible for transduction cascades from the cell surface to the nucleus. TKs are classified as *i*) transmembrane receptor-linked kinases (i.e., RET, vascular endothelial growth factor receptor (VEGFR), epidermal growth factor receptor (EGFR), platelet-derived growth factor receptor (PDGFR), MET, and c-KIT), which are high-affinity cell surface receptors that can be activated by various ligands (i.e., VEGF, EGF, cytokines, and hormones), and *ii*) non-receptor TK, also known as tyrosine phosphatase, referred to as cytoplasmic enzymes involved in transduction cascades (i.e., RAS-RAF-MEK-ERK, PI3K-AKT-mTOR, and JAK-STAT) (14). TK proteins are strongly involved in oncogenesis *via* aberrant expression of their receptors and reduced expression of their modulating enzymes (15). Different genetic alterations can occur, which are almost always mutually exclusive, such as activating mutations/rearrangements in proto-oncogenes; these genetic alterations result in a “gain-of-function” hyperactive kinase, leading to activation of the RAS-RAF-MEK-ERK axis (MAPK-ERK pathway) and PI3K-AKT-mTOR pathway (**Figure 1**). Thus, the constitutive activation of MAPK-ERK and PI3K-AKT-mTOR cascades due to mutational events represents crucial molecular steps in thyroid carcinogenesis and is responsible for uncontrolled, ligand-independent, aberrant growth stimulus to cancer cell growth. The alterations occurring in the MAPK pathway can lead to distinct clinicopathological characteristics, gene expression, and DNA methylation profiles of TC (16).

### 2.1 Tyrosine Kinase Transmembrane Receptors

The proto-oncogene *RET* encodes a membrane TK transmembrane receptor (TK-R) that functions upstream of the MAPK and PI3K-AKT. *RET* is an oncogene that can be overactivated either *i*) by a fusion rearrangement of the TK domain of *RET* gene and the 5′ domain of other genes (known as *RET*/PTC rearrangements) or *ii*) by activating point mutations; *RET* alterations lead to activation of mitogen-activated MAPK pathway, resulting in cancer development. Nikiforov (2002) discussed the most common *RET*/PTC rearrangements occurring in TC: among at least 10 different types of *RET*/PTC, the most common types are *RET*/PTC1 and *RET*/PTC3, accounting for >90% of all rearrangements (17). He reported that the most frequent *RET*/PTC rearrangements occur in a follicular histological type of TC (PTC). Moreover, different types of *RET*/PTC correlated with distinct clinical features of PTC: *RET*/PTC1 leads to a benign clinical course in tumors with typical papillary growth and microcarcinomas, whereas *RET*/PTC3 tends to be associated with the solid variant of PTC and more aggressiveness (17). Instead, activating *RET* point mutations have been exclusively found in MTC: *RET*-activating point mutations are present at the germline level in approximately 100% of hereditary forms and 50% of sporadic cases (4). Thus, tests to identify *RET* germline mutations are required both in hereditary forms and in apparently sporadic cases to identify respectively gene carriers and undiagnosed familial tumors (18, 19).

Additional genomic events occurring in TC are gene amplifications resulting in the gain of genes’ copy number that encodes for TK-R, such as EGFRs, PDGFRs (PDGFR-α and PDGFR-β), VEGFRs (VEGFR1, VEGFR2, and VEGFR3), stem cell factor receptor (c-KIT), and hepatocyte growth factor (HGF) receptor (MET) (20).

Among the growth factors, vascular endothelial growth factor (VEGF) has appeared to be the most prominent due to its involvement in tumor angiogenesis and tumor growth. Indeed, the angiogenesis process becomes pathophysiological when the tumor uses it developmentally. VEGF comprises VEGF-A, VEGF-B, VEGF-C, VEGF-D, and the placental growth factor (PGF), each capable of binding different VEGFRs (21, 22). A study conducted among TC cases with follicular origin highlighted that VEGF expression was more prevalent in PTC (79%) than in FTC (50%) or PDTC (37%); moreover, more than half of these tumors co-expressed the VEGF and its receptors, going in an autocrine loop of VEGF signal (23). VEGF overexpression is a consequence of an overactivation of hypoxia-inducible factor-1 alpha (HIF-1α); this factor is expressed in TC cells (especially in ATC cells), but not in normal thyroid tissues, where it causes intratumoral hypoxia. Upregulation of HIF-1α also correlated with increased transcription of *MET* gene in PTC and ATC and correlated with tumor invasiveness (24). MET is a TK-R, also known as an HGF receptor due to its high affinity for HGF. The induction of MET results in the activation of signals implicated with proliferation, cell survival, cell scattering/migration, and morphogenesis. Deregulated HGF-MET signaling is implicated in oncogenesis, tumor invasiveness, and therapeutic resistance in several cancers, including TC (24).

EGFR appears to be an attractive target for molecular therapy due to its role in the pathogenesis of many types of cancer. Overexpression of EGFR in PTC has been associated with a worse prognosis (25).

### 2.2 *NTRK* Gene Fusion in Thyroid Cancer

The occurrence of *NTRK* fusion in TC is very rare. Fusions involving *NTRK* gene family, including *NTRK1*, *NTRK2*, and *NTRK3*, lead to the constitutive activation of active kinase function due to chimeric rearrangements in tropomyosin receptor kinases (TRKs) A, B, and C, respectively. *NTRK* fusion is an oncogenic drive mutation and occurs in approximately 0.3% of all solid tumors (26). Eight *NTRK* fusion types were identified in TC and seem to be associated with a 100% probability of malignancy (27). *NTRK* fusion is mutually exclusive with other driver mutations, but in rare cases, the co-occurrence of *NTRK* fusions and BRAF^V600E^ mutation has been reported (28, 29) A recent study investigated the impact of *NTRK*-rearranged tumors on the prognosis of different TC types (30). The authors detected *NTRK* rearrangements in 59 of 989 TC tissues, with prevalence in PTC due to high pediatric PTC sample size (n = 104), which are known to harbor *NTRK* fusions with a higher frequency (approximately 20%) (30).

### 2.3 MAPK Axis

Genetic alterations occurring in the MAPK axis play important roles in the development of cancer. Several studies showed that the main activating mutations in these transduction pathways can occur in *RAS* gene or *BRAF* proto-oncogenes (31).

*RAS* mutations might result in the acceleration of mitogenic activity and tumor progression. Incidences of *RAS* mutations correlated with the histological differentiation of TC. Evidence suggests that *RAS* mutations may sustain DTC dedifferentiation into PDTC and ATC (32). *RAS* mutations are one of the most frequent alterations found in FTC (40%–50%) (33) and in about 10% of PTC. Aside from *RET* point mutations in MTC, the only other alterations occurring are H- and K-mutations in RAS (reported in about 17%–80% of RET-negative sporadic MTC) (4).

Mutations in *BRAF* gene lead to the constitutive activation of the BRAF kinase (34). In his review Xing (2005) highlighted that among the known common oncogenic genetic alterations occurring in TC, *BRAF* mutations are the most frequent in PTC, with the prevalence of V600E mutation; BRAF^V600E^ mutation is more frequent in PTC (44% in the studied population) and in PTC-derived ATC tumors (24% of the studied population), compared to other histological subtypes, such as FTC, MTC, or benign thyroid tumors (34). Indeed, the abnormal activation of the MAPK cascade, due to mutations and/or rearrangements in *RET*, *RAS*, and *BRAF* genes, characterizes approximately 70% of PTC cases (31). BRAF^V600E^ is one of the most prevalent alterations in ATC and in PDTC; other frequent oncogene alterations in ATC are *PI3CA*, *PTEN*, *IDH1*, and *ALK* mutations (35). Xu et al. (2020) highlighted that *BRAF*, *RAS*, *TP53*, and *TERT* promoter mutations were the most common mutations that occurred in a cohort of 126 cases of ATC (45%, 24%, 63%, and 75%, respectively); in this study, they also described the very rare occurrence of *NTRK* fusion (3 cases) (36). While mutations of *RET*, *BRAF*, and *RAS* occurring in DTC and MTC are almost always mutually exclusive, ATC is characterized by a higher number of mutations in the same tissue, probably responsible for a more aggressive phenotype (12, 35, 37).

The constitutive activation of receptors or tyrosine phosphatase involved in the MAPK axis leads to activation of the MAPK pathway, which is associated with dedifferentiation, due to inhibition in the expression of thyroid hormone biosynthesis genes, including the NIS and thyroid peroxidase. In 2014, The Cancer Genome Atlas (TCGA) Research Network published a comprehensive characterization of 496 PTC (38). This study highlighted that BRAF^V600E^-mutated PTC, which had the strongest activation of the MAPK pathway, showed the most dedifferentiated state, that is, low expression of some thyroid differentiation genes such as SCLC5A5 gene encoding for the NIS, thyroglobulin, or thyroid peroxidase (39). Therefore, drugs inhibiting one or most of the proteins involved in the MAPK pathway could be useful to improve iodine uptake and allow RAI therapy.

### 2.4 PAX8/PPARγ

While paired box gene 8 (*PAX8*) is a thyroid-specific transcription factor, peroxisome proliferator-activated receptor γ (PPARγ) gene is a transcription factor ubiquitously expressed; their fusion resulting in the *PAX8*/PPARγ oncogene that promotes cell growth reduces rates of apoptosis and allow anchorage-independent and contact-uninhibited growth of thyroid cell lines (40). Moreover, it is associated with tumor multifocality and vascular invasion (41). *PAX8*/PPARγ rearrangements are detected in about one-third of FTC (35%) (40) and a follicular variant of PTC, but not in classic PTC. The major molecular alterations occurring in TC are summarized in **Table 1**.

**Table 1:** Most frequent molecular alterations in various thyroid cancers.

Tumor	Major genetic alterations	Frequency	Reference
**PTC**	VEGF over expression *RET*/PTC rearrangements *- RET*/PTC1 *- RET*/PTC3 *- RET*/PTC2 *BRAF* fusion *RAS* mutations *NTRK* fusion	79% variable depending on geographic region 60–70% of all rearrangements 20-30% of all positive cases <10% 40-50% 10% 20% (in pediatric population)	(23) (17) (37) (30)
**FTC**	VEGF over expression *RAS* mutations *PAX8*/PPAR*γ*	50% 40-50% 35%	(23) (33), (37) (40)
**MTC**	*RET* point mutations RET M918T *RAS (HRAS, KRAS* or *NRAS)*	Approximately 100% of hereditary form 50% of sporadic cases 85% of *RET*-mutated sporadic cases 18-80% of *RET*-negative sporadic form	(4)
**ATC**	*BRAF* ^V600E^ *RAS* mutations *PIK3CA* *PTEN* *Genes in PI3K/AKT/mTOR pathway* *TP53* *NTRK* fusion	45% 24% 18% 10-15% 39% 50-80% rare	(35)
**PDTC**	VEGF over expression *BRAF* mutations *BRAF* ^V600E^ *RAS* mutations Genes in PI3K/AKT/mTOR pathway *TP53*	37% 81% 33% 28% 11% 8-35%	(23) (35)

AKT, alpha serine/threonine-protein kinase; ALK, anaplastic lymphoma kinase; ATC, anaplastic thyroid cancer; BRAF, rapidly accelerated fibrosarcoma kinase; DTC, differentiated thyroid cancer; FTC, follicular thyroid cancer; MTC, medullar thyroid cancer; NTRK, neurotrophic tyrosine receptor kinase; PAX8/PPARγ, paired box gene 8 / peroxisome proliferator-activated receptor γ; PDTC, poorly differentiated thyroid cancer; PTC, papillary thyroid cancer; PTEN, phosphatase and tensin homologous; RAS, rat sarcoma; RET, rearranged during transfection receptor; TP53, tumor protein P53; VEGF, vascular endothelial growth factor.

## 3 Target Therapy in Thyroid Cancer Cases: Focus on (Multi)Tyrosine Kinase Inhibitors

Understanding the molecular pathways involved in the pathogenesis of TC led to the transition of multiple targeted therapies into clinical trials and ultimately into clinical practice (42). Indeed, histological and genomic characteristics play an important role in the prognosis and may guide the treatment of TC. The objective of targeted therapy is to interfere with molecular pathways that are inappropriately activated in cancer cells (33). TKIs are a new class of active compounds that show activity against multiple targets (MTKIs) or a single specific protein (e.g., BRAF inhibitors or *RET*-mutant inhibitors). MTKIs are able to block several TK-Rs, some involved in the pathogenesis of TC (i.e., RET, MET, EGFR, and c-KIT) and others in the vascular angiogenic pathway [i.e., VEGFR1–3 and PDGFR].

In the last few years, several TKIs acting on these molecular pathways have been tested for the treatment of advanced, progressive, and RAI-R DTC, as well as MTC and ATC. TKIs have been shown to improve the PFS of patients in clinical trials as well as in those involved in real-world studies; until 2011, some of them have been approved for use in clinical practice: sorafenib (43, 44) and lenvatinib (45, 46) have been licensed by the Food and Drug Administration (FDA) and European Medicines Agency (EMA) for their clinical efficacy in progressive, locally advanced or metastatic, DTC (papillary/follicular/Hürthle cell), refractory to ^131^I treatment; moreover, vandetanib (47, 48) and cabozantinib (49, 50) have been approved and currently available in patients with metastatic and progressive MTC. In September 2021, cabozantinib also received FDA approval for use in DTC (51). Recently, the FDA approved a high BRAF-specific inhibitor: dabrafenib (52) for BRAF^V600E^-mutated metastatic ATC. Selpercatinib (53) and pralsetinib (54) are the latest approved TKIs by the FDA, occurring respectively in May 2020 and December 2020; these two drugs are indicated for the treatment of *RET*-fusion RAI-R DTC and the treatment of *RET*-mutant MTC in patients aged ≥12 years. EMA approval for the same indications was in December 2020 for selpercatinib, while pralsetinib was approved the last in March 2022.

Larotrectinib and entrectinib are tumor-agnostic TRK inhibitors approved by the FDA and EMA for the treatment of advanced or metastatic solid tumor cancers with *NTRK* fusion (55). All the current approved TKIs for use in the treatment of several types of TC are summarized in **Table 2**.

**Table 2:** Tyrosine kinase inhibitors tested in thyroid cancers: the table summarize molecular targets of each compound and its label for the use in TCs in clinical practice.

Drug	Targets	FDA approval	EMA approval	Clinical indication in TCs treatment
**Vandetanib**	RET, c-KIT, EGFR, VEGFR	04/2011	02/2012	MTC
**Cabozantinib**	RET, MET, c-KIT, VEGFR	12/2012	03/2014	MTC
		09/2021	03/2022	RAI-R DTC, progressed after VEGFR therapy
**Sorafenib**	RET, c-KIT, VEGFR 1-3, PDGFR, BRAF	11/2013	04/2014	RAI-R DTC
**Lenvatinib**	RET, c-KIT, VEGFR 1-3, PDGFR, FGFR	02/2015	05/2015	RAI-R DTC
**Dabrafenib / Trametinib**	BRAF/MEK	05/2018	Not approved for the use in TC	ATC BRAF^v600E^ mutated
**Selpercatinib**	RET	05/2020	12/2020	RET-mutant MTC, RAI-R TC with RET fusion
**Pralsetinib**	RET	12/2020	03/2022	RET-mutant MTC, RAI-R TC with RET fusion
**Larotrectinib**	NTRK	11/2018	07/2019	Advanced solid tumors with NTRK gene fusion
**Entrectinib**	NTRK, ALK, ROS	08/2019	05/2020	Advanced solid tumors with NTRK gene fusion

*Cabozantinib, Lenvatinib and Vandetanib show activity to RET gene fusion yet, not reported in **Table 2**.

ATC, anaplastic thyroid cancer; EGFR, epidermal growth factor receptor; BRAF, rapidly accelerated fibrosarcoma kinase; BRAF^V600E^, valine to glutamic acid substitution of BRAF gene; c-KIT, stem cell factor receptor; DTC, differentiated thyroid cancer; FGFR, fibroblast growth factor receptor; MEK, mitogen-activated protein kinase; MET, hepatocyte growth factor [HGF] receptor; MTC, medullary thyroid cancer; NTRK, neurotrophic tyrosine receptor kinase; PDGFR, platelet-derived growth factor receptor; RAI-R, resistant to ^131^I treatment; RET, rearranged during transfection receptor; ROS, c-ros oncogene 1; VEGFR, vascular endothelial growth factor.

## 4 Management of Tyrosine Kinase Inhibitor Therapy in Thyroid Cancer: Evidence of Efficacy From Clinical Trials

### 4.1 Differentiated Thyroid Cancer

Systemic therapy should be considered in patients with progressive RAI-R DTC. The American Thyroid Association (ATA) guidelines for DTC defined RAI-R DTC as *i*) malignant or metastatic tissues not able to concentrate RAI, *ii*) tissues that have lost the ability to concentrate RAI, *iii*) when metastatic diseases progress despite the ability to concentrate RAI, or *iv*) when RAI has concentrated in some tissues and not others (9). Haugen (1999) reviewed the use of several chemotherapeutic agents for the treatment of RAI-R DTC (e.g., doxorubicin, paclitaxel, bleomycin, cisplatin, carboplatin, methotrexate, melphalan, mitoxantrone, and etoposide), which demonstrated no significant improvement in response rates (RRs) (56). Since traditional cytotoxic systemic chemotherapy has had minimal efficacy in patients with metastatic differentiated thyroid disease, ATA guidelines suggest starting treatment with a kinase inhibitor in RAI-R DTC patients with metastatic, rapidly progressive, symptomatic, and/or imminently threatening diseases not sensible to local control using other approaches (9, 57). Currently approved kinase inhibitors for the treatment of progressive DTC that is refractory to RAI treatment included sorafenib, lenvatinib, and, most recently, cabozantinib (FDA approved only). However, several other commercially available TKIs (e.g., axitinib, pazopanib, motesanib, sunitinib, vemurafenib, and selumetinib) approved for other diseases are currently undergoing clinical trials and are being tested for their use in RAI-R DTC (42).

Optimal management of patients with metastatic DTC requires careful consideration of multiple tumor-associated and patient-related factors within the context of an experienced multidisciplinary team. Optimal decision-making with regard to when to initiate TKI therapy in the metastatic disease setting requires the clinicians and the patients to thoughtfully integrate an understanding of the patients’ symptom burden, tumor progression, and potential tolerance of treatment-related side effects (58).

Moreover, it could be useful, as suggested by Tuttle et al. (2017), to use the doubling time curves to define the critical point in time when the volume and rate of progression of metastatic structural disease deserve consideration for starting a systemic therapy (58).

Patients with disease progression while on initial kinase therapy should be considered for second-line kinase inhibitor therapy. Disease progression was evaluated following Response Evaluation Criteria in Solid Tumors (RECIST) criteria (59).

In addition, as recently shown, re-challenge with lenvatinib could be an option in subjects with metastatic DTC in progression after initial response to the same TKI (60).

#### 4.1.1 Phase II–III Trials in Differentiated Thyroid Cancer

Sorafenib is an oral MTKI targeting VEGFR1–3, PDGFR, RET, c-KIT, and BRAF, currently approved for use in metastatic RAI-R DTC.

Several phase II trials have investigated the efficacy and tolerability of sorafenib in DTC [**Table 3**]. Median PFS ranged from 58 to 84 weeks, partial responses (PR) ranged from 15% to 25% of DTC patients, and stable disease (SD) ranged from 34% to 68% (61–64).

**Table 3:** Phase II trial tested sorafenib in DTC.

Phase II trials of Sorafenib in DTC					
Reference	Patients (n)	Partial Response	Disease stabilization	mPFS (weeks)	Reference
Gupta-Abramson et al., 2008	30	23℅	53%	79	(61)
Kloss et al., 2009	41	15℅	56℅	60	(62)
Hoftijzer et al., 2009	31	25℅	34℅	58	(63)
Ahmed et al., 2011	19	25℅	68%	84	(64)

Sorafenib was approved by the International Regulatory Agencies after a large, phase III study (43). The DECISION trial was an international, multicenter, randomized double-blind, placebo-controlled, phase III study that evaluated the efficacy and safety of sorafenib in patients with progressive RAI-R DTC. A total of 417 patients were randomized to receive sorafenib 400 mg twice daily (n = 207) versus placebo (n = 210). Tumor stratification was 57% PTC, 25% FTC, and 10% PDTC. The primary endpoint was PFS; the median PFS was 10.8 months in the sorafenib group versus 5.8 months in the control arm, showing a significant improvement in the sorafenib group (p < 0.001). OS was not significant across groups, because results in OS in the sorafenib group were affected by the patients in the placebo arm who crossed over to treatment (71%). Its objective RR (ORR) was 12% as compared to the placebo group at 0.5%; the SD rate for more than 6 months was 42% in those who received sorafenib as compared to 33% in the placebo group. Although the independent central review confirmed 12% ORR by RECIST criteria, most patients experienced a reduction in target tumor lesions.

Lenvatinib is an oral inhibitor of VEGFR1–3, PDGFR, FGFR1–4, RET, and c-KIT that has been approved by the FDA and EMA for the treatment of RAI-R DTC.

Initially, the effectiveness of lenvatinib in RAI-R DTC patients was assessed in an open-label, non-comparative, phase II study. In this trial, patients with RAI-R DTC (n = 58) were treated with lenvatinib 24 mg once daily, in a 28-day cycle. Confirmed PRs were observed in 29 patients [RR 50%] (65). Based on these data was planned the SELECT trial, a multicenter, randomized, double-blind, placebo-controlled study testing lenvatinib in RAI-R DTC patients. In the SELECT trial, 392 patients were randomized to receive lenvatinib 24 mg daily in 28-day cycles (n = 261) or placebo (n = 131). At the time of disease progression, patients in the placebo group were receiving open-label lenvatinib. The primary endpoint was PFS. The median PFS was 18.3 months in the lenvatinib group versus 3.6 months in the placebo group. RR was 64.8% versus 1.5% in the placebo group. Median OS was not significant across groups. Four patients in those who received lenvatinib had a complete response (2.4%). The beneficial effect of lenvatinib on PFS was also highlighted in all subgroups of patients. The safety and tolerability of lenvatinib were similar to those of VEGF-VEGFR targeted therapies and mostly manageable. Based on these results, lenvatinib was approved by International Regulatory Agencies for the treatment of RAI-R advanced DTC (45).

It is relevant to point out the differences between the phase III trials (DECISION and SELECT). The first one is the inclusion criteria: previous therapy was not allowed in the DECISION trial, while previous treatment with VEGFR inhibitor was admitted in the SELECT study. Also in the SELECT trial, the progression of disease prior to the inclusion in the trial was established centrally, whereas in the sorafenib trial, it was assessed by investigators. Differences in PFS between the lenvatinib–placebo groups in the SELECT trial were almost three times larger than those in the sorafenib–placebo groups in the DECISION trial. Additionally, the ORR of lenvatinib in the SELECT trial (64.8% in the lenvatinib group versus 1.5% in the placebo group) was more than five times larger than the ORR of sorafenib in the DECISION trial (12.2% in the sorafenib group versus 0.5% in the placebo group). Sorafenib and lenvatinib showed similar toxicity profiles; discontinuation for adverse events (AEs) seems to be equivalent in both trials, but there were six related deaths in the SELECT study compared to only one in the DECISION study (**Table 4**).

**Table 4:** Patients main characteristics and results of SELECT and DECISION trials.

	DECISION	SELECT
**Patient (n)**	416	392
**Arms**	Sorafenib vs placebo	Lenvatinib vs placebo
**Median age**	63 years	64 years
**Histological subtype of DTC [n (%)]** **• Papillary** **• Follicular** **• Poorly differentiated**	118 (57%) 50 (24.2%) 24 (11.6%)	132 (50%) 101(38.7%) 28 (10.7%)
**mPFS (months)**	10.8 vs 5.8	18.3 vs 3.6
**ORR**	12.2% vs. 0.5%, p < 0.0001	64.8% vs. 1.5%, p < 0.001
**OR**	HR 0.80 (95% CI 0.54–1.19) p = 0.14	HR 0.73 (95% CI 0.50–1.07) p = 0.10
**Incidence AE (%)** **(all grade)**	98.6%	97.3%
**Death-related treatment (n)**	1	6
**Reference**	43	45

AE, adverse event; DTC, differentiated thyroid cancer; HR, hazard ratio; mPFS, median progression-free survival; ORR, objective response rate; OS, overall survival.

### 4.2 Medullary Thyroid Cancer

Since the development of MTC is often driven by a single mutation in an oncogenic kinase driver (66), its treatment is different than that of RAI-R DTC.

According to ATA guidelines, treatment with TKIs targeting both RET and VEGFR TK should be considered the treatment of choice in patients with significant tumor burden and advanced MTC. The FDA and EMA have approved vandetanib (2011) and cabozantinib (2012) for the treatment of advanced progressive MTC. Vandetanib was also approved for the treatment of aggressive and symptomatic MTC in children and adolescents aged 5 years and older (67).

#### 4.2.1 Phase III Trials in Medullary Thyroid Cancer

Vandetanib is the “first-in-class” drug approved by the FDA (2011) and EMA (2012) for the treatment of advanced or metastatic MTC (33). Vandetanib is an oral TKI taken once daily and targets several cell receptors, including RET, VEGFR2–3, and EGFR.

A phase III trial (ZETA trial) enrolled 331 patients with advanced (5% of all patients) or metastatic (95%) MTC (47). Patients were randomized to receive vandetanib 300 mg once daily (n = 230) versus placebo (n = 100). All MTC patients could choose to receive vandetanib in an open-label phase. In this study, 56% of patients showed a *RET* mutation, 2% were *RET* wild type, and 41% were unknown. The primary endpoint was PFS. Improvement in PFS was significantly longer in patients treated with vandetanib than in those treated with placebo (30.5 vs. 19.3 months, p < 0.001). OS was not significant across the two arms. There was a good response in patients with M918T-negative tumors and *RET* unknown status. Therefore, the results of the ZETA trial showed that tests evaluating *RET* mutational status are required. Patients with no mutation of RET could not have a benefit from treatment.

These favorable data were conducted for the approval of vandetanib by the FDA for the treatment of advanced MTC (47).

Kreissl et al. (2020) conducted a *post-hoc* analysis of the ZETA trial to assess the efficacy and safety of vandetanib in patients with progressive and symptomatic MTC (48). This subgroup was considered an appropriate representation for the EU label cohort, “aggressive and symptomatic MTC in patients with unresectable locally advanced or metastatic disease” (48). Vandetanib showed statistically significant prolonged median PFS in both the progression and symptoms subgroup and the symptoms-only subgroup, as compared with placebo.

Cabozantinib is a potent inhibitor of several TK-Rs, including VEGFR2, MET, and RET. In a preclinical study, cabozantinib exhibited significant antiangiogenic and antitumor activity in a broad range of tumor models, including a model of MTC with an activating *RET* mutation (68). A phase I dose-escalation study of oral cabozantinib was conducted in patients with advanced solid tumors, with an MTC cohort (37/85 patients) (69). PR was observed in 29% of MTC patients, and 15 of 37 patients with MTC (41%) had SD for at least 6 months. Additionally, the AEs were manageable. According to these results, the safety and efficacy of the drug were evaluated in an international, multicenter, randomized, controlled, phase III trial (EXAM), which enrolled 330 patients with progressive, metastatic MTC (49). Patients were randomized 2:1 to receive cabozantinib 140 mg once daily or a placebo until disease progression or intolerable toxicity. No crossover was allowed at the time of progression. The median age of the patients was 55 years. The main sites of metastatic disease were lymph nodes (79.9%), liver (69.4%), lung (53%), and bone (51.1%), with more than 85% of patients having the involvement of two or more sites.

Approximately 50% of patients were found to be *RET* mutation-positive, with M918T being the predominant *RET* mutation (49).

The primary endpoint was PFS. Secondary endpoints were the evaluation of tumor RR and OS. The main efficacy outcomes measured were PFS, ORR, and response duration. Patients in the cabozantinib group showed a significant improvement in PFS compared with the placebo group (11.2 vs. 4.0 months; p < 0.0001). PRs were observed only among patients in the active treatment arm (27% vs. 0%; p < 0.0001); 47.3% of patients in the cabozantinib group were alive and free of disease progression at 1 year as compared with 7.2% of patients in the placebo group. The median duration of response was 14.7 months.

Subsequently, the authors investigated the association between *RET*/RAS mutational status and PFS/tumor RR in the MTC patients of the EXAM trial. Across the group of patients treated with cabozantinib, those with a *RET* mutation had longer median PFS (60 weeks) than those with wild-type tumors (p = 0.0001). In addition, the patients in which tumors possess a *RET* M918T mutation showed a longer median PFS on treatment than those with any other *RET* mutation (p = 0.009). Patients with hereditary MTC had similar PFS to those with sporadic disease. The RAS-mutated patients showed a similar RR (31%) and PFS (47 weeks) as the *RET*-mutated group (with RR and PFS of 32% and 60 weeks, respectively) (49).

### 4.3 Cabozantinib Approval in Progressed Advanced Thyroid Cancer Resistant to ^131^I Treatment Differentiated Thyroid Cancer

In September 2021, the FDA approved cabozantinib for the treatment of RAI-R DTC that has progressed after previous treatment with a VEGFR inhibitor. This approval is based on the results of the phase III, randomized, double-blind, placebo-controlled clinical trial COSMIC-311, ClinicalTrial.gov ID: NCT03690388 (51). Enrolled patients (n = 187) were randomized 2:1 to receive cabozantinib 60 mg once daily or a placebo. The primary endpoints of the study included PFS and ORR. Cabozantinib showed significant improvement in PFS of 11 versus 1.9 months as compared to the placebo group (hazard ratio (HR), 0.22; 95% CI, 0.15–0.31). Cabozantinib could represent an important treatment option in patients affected by RAI-R DTC who have progressed following prior therapy.

### 4.4 Selective RET Kinase Inhibitors: Selpercatinib and Pralsetinib in RET-Mutant Thyroid Cancer

Selpercatinib and pralsetinib are highly selective RET kinase inhibitors. Approval of both agents provided new treatment options for the patient population with RET-altered TC. Selpercatinib was approved by the FDA and EMA in 2020 for the treatment of RET-mutated MTC and RAI-R RET-fusion TC. The efficacy of selpercatinib in RET-mutated TC was investigated in a phase I–II study (LIBRETTO-001, ClinicalTrials.gov ID: NCT03157128), showing durable efficacy with mainly low-grade toxic effects in patients with MTC who previously received cabozantinib or vandetanib (n = 55), or were treatment naive (n = 88), and in patients harboring RET fusion-positive TC (n = 19) (53). Results of the trial documented high efficacy of selpercatinib associated with minimal side effects and excellent tolerability.

In December 2020, the FDA approved pralsetinib for advanced or metastatic RET-mutant MTC or RET-positive RAI-R TC. In March 2022, EMA approved pralsetinib for the same indications. The efficacy of pralsetinib in patients with RET-altered TC was investigated in a multi-cohort, open-label, phase I/II trial (ARROW, ClinicalTrials.gov ID: NCT03037385) (54). The clinical trial enrolled both patients with RET-mutant MTC (n = 122) who were treatment naive or had previously received cabozantinib or vandetanib, or both, as well as patients with RET-fusion positive TC (n = 20) (RR of 71%, 60%, and 89%, respectively) (54). Pralsetinib had a manageable safety profile in patients with RET-altered TC and provided meaningful clinical activity in patients irrespective of previous treatment history (54).

Although vandetanib and cabozantinib were previously approved for the treatment of metastatic MTC, some patients are not eligible for these therapies on the basis of an unacceptable risk of bleeding or concerns about wound healing (70). Indeed, treatment with both agents required dose reduction in 35% of the patients receiving vandetanib and 79% of those receiving cabozantinib and permanent discontinuation of therapy in 12% and 16% of the patients, respectively observed in the ZETA trial and EXAM trial (47, 49, 53). Until the introduction of selpercatinib and pralsetinib in clinical practice, there were no standard therapies in patients who progressed on cabozantinib or vandetanib (54). Both selpercatinib and pralsetinib showed high activity in patients who previously received cabozantinib or vandetanib, or both (ORRs of 60% and 69%, respectively), including in patients with the gatekeeper V804L/M mutation, which confers resistance to both therapies (53, 54). In the LIBRETTO-001 and ARROW clinical trials, in the arms of patients with treatment-naive RET-mutant MTC who received selpercatinib or pralsetinib, ORRs were 73% (95% CI, 62–82) and 71% (95% CI, 48–89), respectively, suggesting that these RET-targeted inhibitors might have a therapeutic advantage over available first-line MTKIs in the RET-altered population (53, 54). The utility of RET inhibitors in the population of patent with RET fusion-positive TC who previously received RAI was also demonstrated: pralsetinib showed favorable activity in these patients’ ORRs (89%), compared with rates reported in patients with radioiodine-refractory TC treated with sorafenib (12%) and lenvatinib (65%1) (54).

### 4.5 Anaplastic Thyroid Cancer

Since ATC is a rare and aggressive tumor, it is still challenging to predict the patient clinical therapy responsiveness. Until a few years ago, there were no efficient treatments leading to an improvement in survival in those patients. Several genetic mutations have been described in ATC, involved in different molecular pathways linked to tumor progression (shown in **Table 1**). Few clinical trials investigated the use of TKIs in the treatment of ATC (71–74), to improve the quality of life in these patients. Collectively, the results of these studies including target therapy in the treatment of ATC have been overall disappointing. Among these studies, a phase II/III trial (FACT, ClinicalTria.gov ID: NCT00507429) evaluated combretastatin A (CA4P) in combination with carboplatin and paclitaxel in patients with ATC (n = 80) and suggested a survival benefit with the combination therapy. Indeed, the median survival time was 5.2 months in the CA4P arm versus 4.0 months in the control group, showing a reduction in the risk of death of 35%. The 1-year survival was 27% in the CA4P group versus 9% in the control group.

Treatment of ATC has had major advances in the last several years (75). Ferrari et al. (2019) conducted a systematic review of studies involving the treatment of anaplastic thyroid from 1995 to 2017 (76). They highlighted that great attention has been given to the epigenetic alterations underlying thyroid carcinogenesis, including those that drive PDTC and ATC (76, 77). Among these, BRAF^V600E^ is a common somatic mutation occurring in ATC, which can be effectively treated with BRAF/MEK inhibitors (78). Recently, the FDA approved dabrafenib and trametinib in BRAF^V600E^ mutant ATC, based on a phase II trial in which the treatment using these two TKIs showed a remarkable RR of 69% in patients with ATC (n = 16), with one patient achieving complete response (52). *NTRK* fusion also can occur in ATC; rare ATC harboring *NTRK* fusion can be treated with recent approved agnostic therapies (75).

### 4.6 NTRK Inhibitors in Thyroid Cancer

The guidelines of the American Society of Clinical Oncology and European Society for Medical Oncology recommended the use of larotrectinib and entrectinib as treatment of choice for solid tumors with NTRK gene fusions, including TC (79), but not NTRK-mutated, solid tumors (75). Regulatory approval of both agents was based on data from single-arm phase I/II studies, including tumor-agnostic basket trials that enrolled patients based on the presence of NTRK gene fusions (55).

Larotrectinib is an inhibitor of TRK 1–3 and was tested in adult, adolescent, and pediatric patients with solid tumors harboring NTRK gene fusion (a phase I study, ClinicalTria.gov ID: NCT02122913; SCOUT phase 1/2 study, ClinicalTria.gov ID: NCT02637687; NAVIGATE basket study, ClinicalTria.gov ID: NCT02576431) (79). The efficacy of larotrectinib in TC was provided in a recent pooled data analysis derived from three phase I/II clinical trials (ClinicalTria.gov ID: NCT02576431, NCT02122913, NCT02637687) that enrolled 29 patients with TRK fusion-positive TC (80). Patient stratification and the main clinical outcome of this study are shown in **Table 5**.

**Table 5:** Patients main characteristics and results of pooled data from three phase I/II larotrectinib clinical trials (NCT02576431, NCT02122913, NCT02637687).

Reference	79
**Patient (n)**	29
**Median age**	60 years (2 children)
**Histological subtype [n (%)]** **PTC** **FTC** **ATC**	20 (69%) 2 (7%) 7 (24%)
**mPFS**	10.8 vs 5.8
**ORR** **PTC/FTC** **ATC**	71% (95% CI 51-87) 29% (95% CI 4-71)
**TTR**	1.87 months
**mPFS**	69%
**OS**	76%
**AEs**	Grade 1-2
**DCR *(24 weeks)*** **PTC/FTC** **ATC**	91% (95% CI 71-99) 29% (95% CI 4-71)

AE, adverse event; ATC, anaplastic thyroid cancer; CR, complete response; DCR, disease control rate; FTC, follicular thyroid cancer; mPFS, median progression-free survival; ORR: objective response rate; OS, overall survival; PD, progressive disease; PTC, papillary thyroid cancer; TTP, time to treatment failure.

Entrectinib inhibits TRK 1–3, as well as the ALK and ROS1 TKs; its approval was based on a pooled analysis of 3 single-arm phase 1/2 trials that enrolled a total of 54 adult patients with solid tumors harboring NTRK gene fusion (ALKA-372-001, EudraCT: 2012-000148-88, STARTRK-1, ClinicalTria.gov ID: NCT02097810, and STARTRK-2, ClinicalTria.gov ID: NCT02568267) (26). The results of this study highlighted that entrectinib is a safe and active treatment option for patients with *NTRK* fusion-positive solid tumors.

The efficacy and safety of entrectinib and larotrectinib highlighted the need to routinely test for *NTRK* fusions (26).

These tumor agnostic therapies may be relevant also in ATC with RET fusions (75).

### 4.7 No-Label Investigated Use of Tyrosine Kinase Inhibitors in Thyroid Cancer

Sorafenib and lenvatinib have been investigated for use in MTC (81, 82), and a clinical trial was conducted to investigate the use of vandetanib and cabozantinib in RAI-R DTC (51, 83, 84), which led to recent cabozantinib approval as second-line therapy of RAI-R DTC. These studies are summarized in **Table 6**.

**Table 6:** Not-approved use of sorafenib, lenvatinib, vandetanib and cabozantinib.

Drug	Study design / ClinicalTrial.gov ID	Enrolled patients	Treatment arm(s)	Results	Reference
**Sorafenib**	Phase II, open label	Hereditary or sporadic MTC: 16	sorafenib 400 mg orally twice daily	PR: 6.3% SD: 87.6% mPFS: 17.9 months	(81)
**Lenvatinib**	Phase II, multicenter, open-label, single-arm [NCT00784303]	MTC: 59	lenvatinib 24 mg orally once daily	ORR: 36% DCR: 80% SD: 44% mPFS: 9 months	(82)
**Vandetanib**	Phase II, randomized, Double-Blind, Placebo-Controlled [NCT00537095]	DTC (advanced or RAI-R): 145	vandetanib 300 mg per day (n=72) vs placebo (n=73)	mPFS: 11.1 (vandetanib) vs 5.9 (placebo) months	(83)
	Phase III, randomized, Double-Blind, Placebo-Controlled, Multi-Centre [NCT01876784]	DTC (advanced or RAI-R): 238	vandetanib 300 mg once daily vs placebo (119 patients for each group)	N/A Estimated end date 12/2022	
**Cabozantinib**	Phase II, single arm, open label [NCT02041260]	RAI-R DTC: 35	cabozantinib 60 mg orally once a day	PR: 54% SD: 43% durable SD (≥6 months): 26% mPFS not reached	(84)

DCR, disease control rate; DTC, differentiated thyroid cancer; MTC, medullary thyroid cancer; N/A, not reported; ORR, objective response rate; PFS, progression-free survival; PR, partial response; RAI-R, resistant to ^131^I treatment; SD, stable disease.

Several other TKIs approved for the treatment of other tumors by national and international regulatory agencies have been extensively investigated in clinical trials for the treatment of all types of TC (e.g., axitinib, pazopanib, motesanib, sunitinib, vemurafenib, and selumetinib). These trials, previously reviewed by other authors in the last years (41, 85, 86), are summarized in **Table 7**.

**Table 7:** Phase II-III trials investigated target therapy in all type of TCs.

Drug	Targets	Study design / ClinicalTrial.gov ID	Enrolled patients and timing	Treatment arm(s)	Results	Reference
**Selumetinib**	MEK	Pilot study, single arm [NCT00970359]	Metastatic TC: 20 (enrolled from 08/2010 to 12/2011)	75 mg selumetinib twice daily. Within 1 month, patients with adequate RAI uptake may receive 131I per standard of care and continue selumetinib until 2 days following 131I.	12/20 increased the uptake of iodine-124 PR: 5/20 SD: 3/20	(87)
		Phase II, single arm, open label [NCT00559949]	RAI-R PTC: 39 (enrolled from 12/2007 to 06/2009)	100 mg selumetinib twice daily	PR: 3% SD: 54% PD: 28% mPFS: 32 weeks	(88)
		Phase III, randomized, double blind (ASTRA) [NCT01843062]	DTC at high risk of primary treatment failure: 233 (enrolled from 08/2013 to 03/2016)	Selumetinib 75 mg BD + RAI	CR: 40%	(89)
**Pazopanib**	VEGFR, PDGF, c-KIT	Phase II [NCT00625846]	RAI-R DTC: 39 (enrolled from 02/2008 to 01/2009)	800 mg pazopanib once daily	PRs: 49% FTC PRs: 73% PTC PRs: 33%	(90)
		Phase II [NCT00625846]	RAI-R DTC: 60	800 mg pazopanib once daily	PRs: 36.7%	(91)
		Phase II PAZOTHYR [NCT01813136]	RAI-R TC: 168 (enrolled from 06/2013 to 01/2018)	800 mg pazopanib once daily (n=100 were randomized 1:1 to receive continuos [CP] vs intermittent [IP] pazopanib treatment)	IP TTP: 14.7 CP TTP 11.9 (p=0.35)	(92)
		Phase II, multicenter (enrolled from 09/2008 to 12/2011) [NCT00625846]	MTC: 35 (enrolled from 09/2008 to 12/2011)	800 mg pazopanib once daily	PR: 14.3% PFS: 9.4 months OS: 19.9 months	(93)
		Phase II, multicenter, single arm [NCT01236549]	ATC: 16 (enrolled from 02/2008 to 02/2011)	800 mg pazopanib once daily	No confirmed RECIST responses	(71)
**Sunitinib**	VEGFR, PDGFR; RET, c-KIT, FLT	Phase II single center, nonrandomized, open-label [NCT00668811]	Advanced DTC: 23 (enrolled from 09/2008 to 02/2015)	37.5 mg sunitinib once daily	ORR: 26% mPFS: 241 days	(94)
		Phase II, single arm, open label (THYSO) [NCT00510640]	RAI-R DTC, MTC or ATC: 71 [DTC: 41, ATC: 4, MTC: 26] (enrolled from 08/2007 to 10/2009	50 mg sunitinib once daily	DTC ORR: 22% MTC ORR: 38.5% ATC ORR : not observed	(72)
		Phase II, single center	RAI-R DTC or MTC: 35 [DTC: 28 MTC: 7] (enrolled from 08/2007 to 02/2009)	37.5 mg sunitinib once daily	ORR: 31%	(95)
**Motesanib**	VEGFR 1-3, PDGFR, c-KIT, RET wild type	Phase II, open label [NCT00121628]	RAI-R DTC: 93 MTC: 91 (enrolled from 07/2005 to 03/2006)	125 mg motesanib once daily	DTC ORR: 14% DTC SD: 67% (maintained for 6 months in 35% of patients) MTC ORR: 2% MTC SD: 81% MTC durable SD (≥24 weeks): 48%	(96) (97),
**Apatinib**	VEGFR-2	Phase II [NCT02731352]	RAI-R DTC: 20 (enrolled from 03/2016 to 02/2021)	500 or 750 mg apatinib once daily	ORR: 80% DCR: 95%	(98) (99),
		Phase III, exploratory, randomized, double arms [NCT03048877]	RAI-R DTC: 92 (enrolled from 09/2017 to 08/2020)	500 mg apatinib once daily vs placebo	mPFS: 22.2 months	(100)
**Axitinib**	VEGFR	Phase II [NCT00389441]	RAI-R DTC or MTC : 52 (enrolled from 12/2006 to 09/2008)	5 mg axitinib twice daily	ORR: 35%	(101)
		Phase II, single arm [NCT00094055]	RAI-R TC: 60 [DTC: 45 MTC: 11 ATC: 2 Other: 2]	5 mg axitinib twice daily	ORR: 30% PFS: 18.1 months [MTC PRs: 18% MTC SD: 27%]	(73) (74),
			RAI-R DTC or MTC: 47 (enrolled from 10/2012 to 11/2014)	5 mg axitinib twice daily	mORR: 27,7% (DTC ORR:29.4%; MTC ORR: 23.1%)	(102)
**Donafenib**	VEGFR, PDGFR, RAF	Phase II [NCT02870569]	RAI-R DTC: 35	300 mg donafenib twice daily (17) or 200 mg twice daily (18)	200 mg group ORR: 12.5% 300 mg group ORR: 13.33%	(103)
		Phase III, randomized [NCT03602495]	RAI-R DTC: 204	Patients were randomized 2:1 to receive 300 mg donafenib twice daily vs placebo	N/A	
**Dovitinib**	VEGFR, FGFR	Phase II	TC: 40 [DTC: 28; MTC: 12] (enrolled from 01/2013 to 10/2014)	Dovitinib 500 mg once daily for five consecutive days, followed by a 2-day rest every week	ORR: 20.5% PFS: 5.4 months OS not reached	(104)

ATC, anaplastic thyroid cancer; c-KIT, stem cell factor receptor; CR, complete response; DTC, differentiated thyroid cancer; FGFR, fibroblast growth factor receptor; MTC, medullary thyroid cancer; N/A, not reported; ORR, objective response rate; OS, overall survival; PDGFR, platelet-derived growth factor receptor; PFS, progression-free survival; PD, progressive disease; PRs, partial responses; RAF, rapidly accelerated fibrosarcoma; RAI-R, resistant to ^131^I treatment; RECIST, response evaluation criteria in solid tumors; RET, rearranged during transfection receptor; TTP, time to treatment failure; VEGFR, vascular endothelial growth factor.

## 5 Adverse Events Associated With Tyrosine Kinase Inhibitors and Their Management

Currently, treatment of TC is based on the use of TKIs. Nevertheless, target therapy shows some limitations due to the development of AEs or the development of tumor resistance mechanisms. Adequate management of side effects is important to minimize toxicities: thus, patients can continue therapy and obtain a response in terms of survival prolongation (57, 105). Although the use of TKIs has largely avoided the common toxicities associated with the use of chemotherapeutic agents (e.g., alopecia, nausea, and vomiting), these drugs present particular side effects depending on their on-target and off-target effects. Side effects can be “on-target,” related to exalted effect at the target, or “off-target,” when they result from the modulation of other targets that could be biologically or totally unrelated to the target (14, 106). The most common side effects related to treatment with TKIs include hepatic impairment, gastrointestinal events, hypertension, proteinuria, and fatigue. AEs frequently occur in about 2–3 weeks after the start of drug treatment and influence the adherence of patients to the treatment. AEs rarely are severe and life-threatening. Moreover, TKIs consist of pills taken daily at patients’ homes for a long time, until progression or unacceptable toxicity. New challenges in the treatment approach using TKIs are a balance between inhibition of oncogenic driver kinase activity and drug side effect management in order to achieve an optimization of patients’ quality of life. In this context, in order to manage early toxicities related to drugs, both patients and physicians should be educated to recognize them.

The latest TKIs approved (selpercatinib and pralsetinib) showed a better toxicity profile as compared to other MTKIs. In the ARROW study, pralsetinib was well tolerated with a predictable safety profile, and the rates of dose reductions and treatment discontinuations because of treatment-related AEs were low when compared with available MTKIs (54).

The major AEs associated with TKIs used in TC will be discussed, as follows (**Table 8**) (107).

**Table 8:** Major AEs (> grade 3) associated with available TKIs approved for the use in TC.

	Vandetanib	Cabozantinib	Sorafenib	Lenvatinib	Selpercatinib	Vandetanib
**Hepatic impairment** **ALT** **ASP**					11% 9%	
**Diarrhea**	11%	15.9%	2%	8%	6%	
**Hypertension**	9%	8.4%	4%	41.8%	21%	17%
**ECG QT prolonged**	8%					
**Proteinuria**		0.9%		10%		
**Hand foot syndrome**		12.6%	6%	3.4%		
**Fatigue**	13%	9.3%	5%	9.2%		
**Other AEs**	Decreased appetite: 4% Rash: 8% Asthenia: 3%	Mucosal inflammation: 2.8% Hypocalcemia: 2.8%		Thromboembolic effects: 3.8%		Neutropenia: 13% Lymphopenia: 17% Anemia: 10%
**Discontinuous therapy**		n=35/214 16%		n=37 (14.2%)	n=12/531 (2%)	n=5/142 (4%)
**Death**				n=6		n=1
**Reference**	(47)	(49)	(43)	(45)	(53)	(54)

### 5.1 Hepatic Impairment

TKIs can cause liver injury. Currently, the mechanism underlying hepatotoxicity is partially unknown but probably is not related to kinase inhibition in hepatocytes (108). TKI-associated hepatotoxicity may not only be due to the parent drug but also due to metabolites produced from the metabolism of cytochrome P450 3A2 (109, 110).

The onset of TKI-induced hepatotoxicity usually appears within the first 2 months of starting treatment but could be delayed and is usually reversible. Fatality from TKI-induced hepatotoxicity is uncommon but requires diligent surveillance (108).

The first clues of hepatotoxicity occurrence are vague symptoms such as fatigue, anorexia, nausea, discomfort in the right upper quadrant, and dark urine (111). Biochemical markers of liver injury include elevation of alanine aminotransferase (ALT), aspartate aminotransferase (AST), alkaline phosphatase (ALP), and bilirubin, as well as an alteration in protein synthesis, which is reflected in albumin concentration and time of prothrombin (PT) (108).

Ghatalia et al. (2014) conducted a meta-analysis of randomized controlled trials to determine the RR of hepatotoxicity with VEGFR TKIs (112). High-grade ALT elevation occurred in 168 of 9,930 (1.7%) patients and AST elevation in 159 of 9,986 (1.6%) patients receiving VEGFR TKI. The incidence of high-grade liver failure/dysfunction was 0.7% (112).

In the phase I/II LIBRETTO-001 trial, approximately one-half of treated patients had elevations in transaminases during treatment with selpercatinib; they were G3 or G4 in approximately 10%. The median time to onset was approximately 4 weeks. Elevations in total bilirubin occurred in approximately one-fourth of treated patients; they were G3 or G4 in 2% (113).

In the phase I/II ARROW study (n = 438), pralsetinib-treated patients were reported to have increased AST and ALT levels in 34% and 23% of the cases, respectively (114).

Although the incidence of life-threatening liver failure reported with VEGFR TKIs is quite small, careful monitoring of hepatic function and exclusion of patients with moderate hepatic impairment may be essential in patients receiving VEGFR TKIs. Patients treated with any of these agents should have a baseline evaluation of liver function tests and periodic re-evaluation during therapy.

### 5.2 Gastrointestinal Toxicities

Gastrointestinal toxicities of TKIs include diarrhea, nausea, vomiting, and, in some cases, pancreatic atrophy.

In clinical trials, nausea of any grade has been reported in 23% to 58% of treated patients, and rates are the highest in patients treated with lenvatinib, cabozantinib, and selpercatinib (45, 49, 113). Vomiting of any grade has been reported in a range of 10% to 48% of treated patients; rates are the lowest with pazopanib and sorafenib and the highest with lenvatinib and cabozantinib (49, 106, 113).

Diarrhea is a common side effect related to TKIs. In clinical trials, diarrhea of any grade has been reported in a range of 30% to 79% of patients treated with TKI (highest rates with vandetanib). Severe forms of diarrhea (G3 or G4) occurred in 3% to 17% (115). Vandetanib acts as a moderately potent EGFR and VEGFR inhibitor; its mechanism of action explains the somewhat higher incidence of diarrhea associated with this agent (83).

In the case of G1–G2, diarrhea may improve with loperamide; if it persists, a coproculture test and antibiotic therapy are useful, followed by hydration and octreotide administration.

If antidiarrheal therapies are not sufficient, a dose reduction or discontinuation of TKI therapy could be necessary. In the case of severe toxicity, it could be necessary to restart TKI therapy at a reduced dose. It is suggested to take the medication with a large meal and plenty of water to reduce side effects (107).

Long-term therapy with sorafenib is associated with pancreatic atrophy. The possibility of pancreatic exocrine insufficiency should be considered in patients treated with sorafenib who develop refractory diarrhea.

### 5.3 Cardiovascular Effects

TKIs are associated with cardiovascular toxicity, due to the involvement of TK-R in normal cellular homeostasis. Common cardiovascular events are hypertension, reduced ejection fraction, myocardial infarction, and QT prolongation. Clinical drug-by-drug cardiotoxicity incidence of TKIs has been extensively reviewed by Jin et al. (2020) (116).

#### 5.3.1 Hypertension

Hypertension is a frequent AE related to the use of angiogenesis inhibitors, depending on the blocking of VEGF action in normal physiology.

VEGF inhibition is associated with decreased production of nitric oxide (NO) in the wall of vessels. Vasoconstriction is caused by lower production of NO: this lower production causes an increase in peripheral resistance and blood pressure (117). It has been suggested that the mechanism of hypertension is based also on elevated fluid retention and disruption of the endothelium (118).

In the phase III study SELECT, hypertension occurred in 73% of lenvatinib-treated patients compared with 15% of placebo-treated patients (119).

Hypertension can occur any time after the start of therapy. Antihypertensive drug treatment could be helpful for its management during treatment with TKIs. The main classes of drugs include ACE inhibitors, calcium channel blockers (CCBs), angiotensin II receptor blockers (ARBs), beta-blockers, and diuretics. ACE inhibitors represent the first step of treatment. In case of inadequate control of blood pressure, a dose increase is requested and the addition of other drugs such as diuretics, beta-blockers, or CCBs. Cardiovascular conditions and renal dysfunction may influence the choice of treatment.

Blood pressure <140/90 is considered adequate. Once treatment with a TKI is started, blood pressure needs to be measured within 1 week. Daily monitoring of blood pressure is requested, and if necessary, antihypertensive treatment should be rapidly titrated or new drugs added to the regimen (117). Blood pressure elevation related to TKI is reversible; thus, discontinuation or dose reduction of TKI can also be used to control hypertension. Discontinuation is useful when the control of symptoms is tough; in this case, after stopping treatment, the patients should be readmitted at the previous treatment with the same dose; in other cases, the dose will be scaled down until blood pressure control is obtained (120).

#### 5.3.2 Other Cardiovascular Effects

Decreases in left ventricular function can be seen in patients treated with any of the VEGF-targeted therapies. A total of 29,252 patients from 71 randomized controlled trials were included in a meta-analysis of VEGFR TKIs, which demonstrated a higher cardiac ischemia relative risk (RR 1.69, 95% CI, 1.12–2.57), with the highest risks observed for sorafenib (121). Left ventricular systolic dysfunction was also increased after VEGFR TKIs (RR 2.53, 95% CI, 1.79–3.57), with the highest risks observed for sunitinib (121).

History of hypertension and coronary artery disease is assumed to increase the risk of cardiotoxicity.

Less data on cardiotoxicity are available with sorafenib (122, 123), but the risk seems to be lower than with sunitinib (about 2%–3%).

In a placebo-controlled trial in patients with advanced TC treated with lenvatinib, cardiac dysfunction (decreased ejection fraction, cardiac failure, or pulmonary edema) was reported in 7% versus 2% in the placebo group (121). Similar data were found in patients treated with pazopanib (124).

A baseline assessment of left ventricular systolic dysfunction could be useful in all patients receiving these drugs.

These drugs delay cardiac repolarization, an effect that is reflected on the surface ECG by a prolonged heart rate corrected QT (QTc) interval. A prolonged QTc interval can be associated with potentially fatal cardiac arrhythmias. The ventricular tachyarrhythmia most typically triggered is of a unique form, known as torsades de pointes, which is usually associated with a fatal outcome.

Patients with a history of QT prolongation or on treatment with antiarrhythmic agents need particular attention. The US Prescribing Information recommends monitoring ECG in patients with congenital long QT syndrome, heart failure, or bradycardia or those taking drugs known to prolong the QT interval.

QTc prolongation is a very important AE in vandetanib and lenvatinib treatment; torsades de pointes and sudden death have been reported in vandetanib-treated patients (125). In the ZETA trial, 14% of patients who received vandetanib showed QTc > 500 ms. Vandetanib is not indicated in patients with QTc > 450 ms (USA) or 480 ms (EU) (124, 126). Thus, cabozantinib may be preferred in patients with preexisting cardiac conditions or patients who are unable to comply with the cardiac monitoring parameters required in this warning (127). Before starting vandetanib, it is helpful to perform ECG and echocardiogram; during vandetanib treatment, it is necessary not to use drugs that prolong QTc and maintain electrolyte levels and serum thyroid-stimulating hormone (TSH) levels in the normal range (128).

QT interval prolongation and cardiac dysfunction are the two main cardiac AEs observed in lenvatinib-treated patients: QTc interval prolongation occurred in 9%, 11%, and 8% of lenvatinib treated arms in the SELECT trial, Study 205, and REFLECT trials, respectively (116); QTc interval prolongation of greater than 500 ms was observed in 2%, 6%, and 2% of patients, respectively (129). As suggested by Reed et al. (2020), the higher-risk patients should undergo echocardiography and cardiac troponin and natriuretic peptide measurement at baseline and should be monitored *via* echocardiograms at each treatment with lenvatinib (130).

### 5.4 Proteinuria

Incidences of proteinuria of any grade during TKI treatment correlate with VEGFR inhibition; thrombotic microangiopathy and acute interstitial nephritis are common with sorafenib. In the SELECT trial, 10% of lenvatinib-treated patients experienced grade 3 proteinuria. While VEGF is present on glomerular podocytes, VEGFR is present on endothelial cells of the glomerulus. The alteration of VEGF and VEGFR mechanisms leads to capillary endotheliosis and proteinuria. Deprivation of endothelial fenestrations in capillaries, an increase of endothelial cells, and deprivation of podocytes inside the glomerulus are a consequence of depletion of VEGF in podocytes. The lack of interaction between podocytes and endothelium maltreats the filtration barrier, leading to proteinuria.

Monitoring of proteinuria is based on a dipstick test, which should be done before initiation of TKI and then every 14 days during treatment. In case of the absence of proteinuria, a test can be performed every 28 days. When proteinuria is G2 or higher, it is useful to evaluate the protein/creatinine ratio (PCR) using a specimen of urine collected in a universal container early in the morning. An alternative test to PCR is the albumin/creatinine ratio (ACR).

G2 proteinuria matches with PCR of 100–300 mg/mmol or ACR of 70–250 mg/mmol. G3 proteinuria matches with PCR > 300 mg/mmol or ACR > 250 mg/mmol. Treatment of high blood pressure with drugs such as ACE inhibitors or ARB can be useful in the management of proteinuria.

### 5.5 Hand-Foot Syndrome

**Figure 2:**
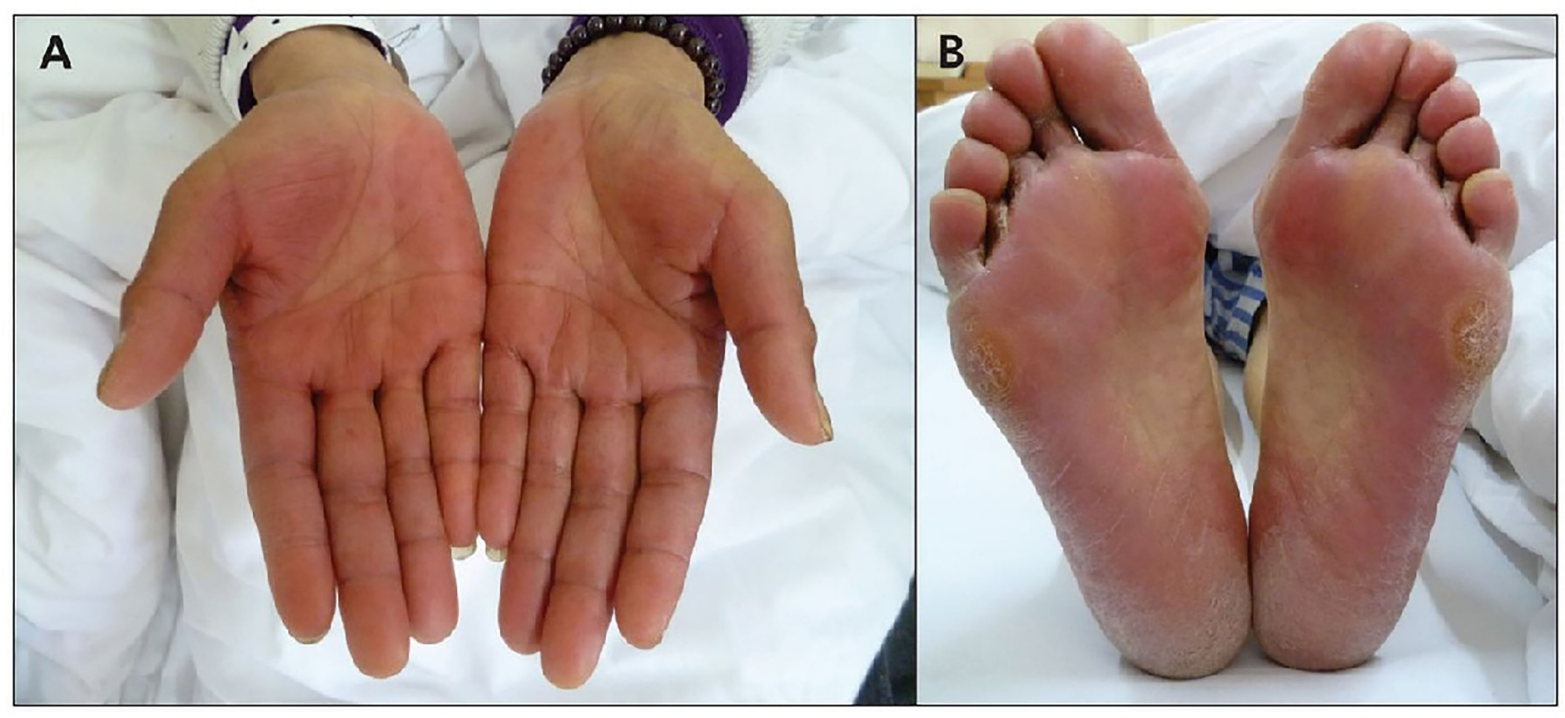
Palma-plantar erythodysesthesia in a patient treated with sorafenib. **(A)** hand syndrome, **(B)** plantar syndrome.

Hand-foot syndrome occurs commonly during therapy with sorafenib or sunitinib (**Figure 2**). These drugs probably impact the growth of skin cells and capillaries of the hands and feet.

Symptoms include redness, swelling, tenderness, and tightness of the skin. When the syndrome is severe, peeling skin, ulcers, and intense pain leading to the limitation in the use of the hands or walking are common. Generally, hand-foot syndrome appears in the 1st to 6th week of treatment.

Prevention measures include limiting exposure to hot water, avoiding contact with chemicals used in cleaning products, and avoiding activities that cause friction on the hands or feet.

Treatment consists of *i)* topical anti-inflammatory medications, such as corticosteroid creams; *ii)* topical anesthetics, which are available as creams or patches to apply on palms or soles in the presence of pain; *iii)* topical moisturizing and exfoliating creams containing urea; *iv)* pain relievers.

In addition, when the syndrome is severe enough to affect patients’ quality of life, dose reduction or drug discontinuation is necessary until symptoms of hand-foot syndrome will improve (131).

Sorafenib is associated with hand-foot syndrome in 30% of patients. In case of a G1 toxicity, it is suggested to continue sorafenib and use topical therapy. In case of a G2 toxicity, it is suggested to use topical therapy; if no improvement within 7 days will be seen, treatment should be interrupted until toxicity resolves to grade 0–1. If G2 or G3 toxicity recurs, it can be useful to decrease the sorafenib dose by one dose level; in case of a 4th occurrence, it will be appropriate to discontinue therapy (132).

### 5.6 Fatigue

Fatigue is a common side effect related to TKIs. The etiology is complex. It can depend on cardiac dysfunction, renal dysfunction, or gastrointestinal side effects, such as diarrhea or nausea. It is very frequent in patients treated with motesanib and axitinib (133). Supportive care is necessary and includes adequate nutrition, exercise, and stress-reducing techniques (107).

Thyroid function should be monitored in case of asthenia in patients receiving TKI therapy.

During TKI therapy, thyroid function abnormalities have been observed in athyrotic patients on thyroxine substitution.

The mechanism of worsening hypothyroidism in thyroidectomized patients is still unclear. These drugs can cause an alteration in the transport and metabolism of thyroid hormones. In particular, sunitinib and sorafenib increase the activity of type 3 deiodinase (as evidenced by the decrease in T3/T4 and T3/rT3 ratios) resulting in hypothyroidism because of lower tissue availability of the active hormone T3, locally inactivated in T2 or rT3 (134).

During the treatment with TKIs, it is required to monitor TSH levels monthly and to adjust thyroid replacement medication as needed. If patients present severe chronic asthenia, primary adrenal insufficiency (PAI) should be excluded. A frequent occurrence of PAI has been reported after the first month of treatment with lenvatinib or vandetanib in patients with advanced TC. Importantly, replacement therapy is associated with substantial relief of these major AEs (135).

The mechanisms involved in the development of lenvatinib-induced PAI are unknown. It was hypothesized that the target of lenvatinib (in particular, VEGFRs and PDGFRα) may be implicated in adrenal control (136).

## 6 Conclusion

Treatment of TC has changed in the last few years. The analysis of the molecular basis of TC guided the development of novel therapeutics. Therapy using TKIs represents an important option *i*) for first-line treatment, *ii*) for the succeeding treatments after the first, *iii*) for the treatment of cancer types that became resistant to other treatments, and *iv*) for the treatment of rare cancer isotype with no chance of cure. Several TKIs have been studied and approved, leading to an improvement in RR and survival in populations affected by various TC types.

The correct management of potential AEs of these drugs remains a goal of interest. We have reported some practical suggestions to better control them.

## Author Contributions

IP and SM wrote the manuscript and designed some tables. FE wrote the manuscript and designed the figures and most of the tables. AP, DS, and MV contributed to the writing of the manuscript. DG made a critical revision of the manuscript. All authors have read and agreed to the published version of the manuscript.

## Conflict of Interest

The authors declare that the research was conducted in the absence of any commercial or financial relationships that could be construed as a potential conflict of interest.

## Publisher’s Note

All claims expressed in this article are solely those of the authors and do not necessarily represent those of their affiliated organizations, or those of the publisher, the editors and the reviewers. Any product that may be evaluated in this article, or claim that may be made by its manufacturer, is not guaranteed or endorsed by the publisher.
